# Isolation and characterization of a novel parvovirus from a red-crowned crane, China, 2021

**DOI:** 10.1186/s12917-023-03683-4

**Published:** 2023-09-21

**Authors:** Hao Liu, Jie Huang, Zi-Shuo Lu, Li-Xia Li, Xiao-Tong Liang, Tian Tang, Wen-Chao Sun, Hui-Jun Lu, Ning-Yi Jin, Xue Bai, Xing-Kui Si

**Affiliations:** 1 School of Life Sciences and Engineering, Foshan University, Foshan, Guangdong Province China; 2 Institute of Virology, Wenzhou University, 325035 Wenzhou, China; 3 Institute of Military Veterinary, Academy of Military Medical Sciences, Changchun, Jilin Province China; 4 Institute of Special Economic Animal and Plant Sciences, Chinese Academy of Agricultural Sciences, Changchun, Jilin Province China; 5 Key Laboratory of Special Animal Epidemic Disease of Ministry of Agriculture and Rural Affairs, Institute of Special Animals and Plants, Chinese Academy of Agricultural Sciences, No. 4899 Juye Street, 130112 Changchun, Jilin province China

**Keywords:** Enterovirus, Red-crowned crane, Parvovirus, Detection, Genome analysis, Phylogeny

## Abstract

**Background:**

Parvoviruses are icosahedral, nonenveloped viruses with single-stranded DNA genomes of approximately 5 kb in length. In recent years, parvoviruses have frequently mutated and expanded their host range to cause disease in many wild animals by altering their tissue tropism. Animal infection mainly results in acute enteritis and inflammation of other organs. In this study, we used a viral metagenomic method to detect a novel parvovirus species in a red-crowned crane that died due to severe diarrhea in China.

**Results:**

The presence of the viral genome in the kidney, lung, heart, liver, and intestine were confirmed by PCR. Histopathological examination of the intestine showed a large number of infiltrated inflammatory cells. The JL21/10 strain of the red-crowned crane parvovirus was first isolated from the intestine. Whole-genome sequence analysis showed that JL21/10 shared high identity with the red-crowned crane Parvovirinae strains yc-8 at the nucleotide level (96.61%). Phylogenetic analysis of the complete genome and NS1 gene revealed that the JL21/10 strain clustered with strains in chicken and revealed a close genetic relationship with the red-crowned crane parvovirus strains.The complete of VP2 gene analysis showed that JL21/10 shared identity with the red-crowned crane yc-8 strains (97.7%), chicken (55.4%),ducks(31.0%) and geese(30.1%) at the amino acid level. The result showed that red-crowned crane parvovirus may be cross-species transmission to chicken. However, There is little possibility of transmission to ducks and geese.

**Conclusion:**

This is the first isolation and identification of a parvovirus in red-crowned crane that was associated with severe diarrhea.

**Supplementary Information:**

The online version contains supplementary material available at 10.1186/s12917-023-03683-4.

## Introduction

Parvoviruses are icosahedral, nonenveloped viruses with single-stranded DNA genomes of approximately 5 kb in length [1]. At present, parvoviruses cause infections worldwide and naturally infect a wide range of hosts. Porcine parvovirus (PPV), canine parvovirus (CPV), feline parvovirus (FPV), goose parvovirus (GPV), duck parvovirus (MDPV), and chicken parvovirus (ChPV) are the most widespread infectious parvoviruses. PPV can cause reproductive disorders in sows, piglet diarrhea, dermatitis and respiratory diseases, causing considerable economic losses to the pig industry. CPV and FPV mainly infect canines and cats and cause severe diarrhea. GPV, MDPV and ChPV are parvoviruses that mainly infect poultry. They can cause goose parvovirus disease, duck parvovirus disease and chicken runting stunting syndrome, which are the main infections that seriously endanger poultry breeding [2].

In recent years, parvoviruses have frequently mutated and expanded their host range to cause disease in many wild animals by altering their tissue tropism. Animal infection mainly results in acute enteritis and inflammation of other organs [3, 4]. However, there are few reports on parvovirus infection in wild birds.

## Materials and methods

### Case report

In October 2021, an adult wild red-crowned crane was admitted for clinical treatment at the Wildlife Rescue and Rehabilitation Center in Jilin Province, China. The animal presented with decreased food intake and bloody stools, lost weight over 5 days of treatment and experienced sudden death (Technical Appendix Figure S1).

### Pathogen examination

We collected tracheal, kidney, liver, esophageal, intestinal and heart tissues to detect the pathogen in the red-crowned crane. To identify possible causes of illness, the tissue samples with clinical symptoms were pooled for viral metagenomic analysis as previously described [5]. In addition, The DNA and cDNA were subjected to PCR to detect a panel of potential viral pathogens, including avian Influenza virus (AIV) [6], Newcastle disease virus (NDV)[7], GPV, MDPV [8] and ChPV [9]. The FastPure Viral DNA/RNA Mini Kit (Vazyme Biotech Co., Ltd., China) was used for RNA extraction. The RNA was converted to cDNA using a Vazyme HiScript II 1st Strand cDNA Synthesis Kit (Vazyme, China) in accordance with the manufacturer’s instructions.

### Distribution of the virus in organs, and histopathological examination

To examine the distribution of parvovirus in the infected red-crowned crane, specific primers RCCPV-F0 and RCCPV-R0 were designed for polymerase chain reaction (PCR) according to the matching positions in the sequence assembly (Table 1). Tracheal, kidney, liver, esophageal, intestinal and heart tissues were collected for PCR detection of the parvovirus. Samples of intestinal tissues were subjected to hematoxylin and eosin staining.

**Table 1:** Oligonucleotide sequences of primers used in study of a novel parvovirus isolated from a red-crowned crane, China, in 2021

Primer	Oligonucleotide sequence,5’→3’	Reference	Length
RCCPV-F0	AGGGTGGAGCTAATGGATAATG	Designed for this study	653 bp
RCCPV-R0	GACGTGAACCCGGAGATAAA		
RCCPV-F1	CAGCTGTCTGGCGACTGAGG	Designed for this study	2646 bp
RCCPV-R1	AATCCCGTTACACCCGTCCG		
RCCPV-F2	ACAACGGCAACTTCCCGTTTAA	Designed for this study	970 bp
RCCPV-R2	CAGGCTAGGATCCACAACGC		
RCCPV-F3	CTGACCTCTGAGGCCGACTC	Designed for this study	586 bp
RCCPV-R3	TCGTAAGGCGTTCTGAACCC		
RCCPV-F4	GATACAGCAAATAGATGGGT	Designed for this study	958 bp
RCCPV-R4	ATCTCTAGTCAGACACACGC		
RCCPV-F5	GAGAGCACGGGGAACTGGAC	Designed for this study	1555 bp
RCCPV-R5	ATTTATATAATTACACAGCCC		

### Isolation and genetic analysis of the virus

To identify the causative pathogen, the supernatants of intestinal tissues from the red-crowned crane were injected the allantoic cavity of 9-day-old specific pathogen-free (SPF) chicken embryos. The total allantoic fluid was diluted 20-fold in DMEM before inoculation onto DF-1-cell monolayers. The cell lines were cultured in DMEM supplemented with 10% fetal bovine serum at 37 °C in a 5% CO_2_ incubator. Cultures were freeze-thawed three times and centrifuged at 4,000×g for 5 min. The clarified supernatants were then passaged in fresh DF-1 cells. Culture supernatants were collected after five passages and stored at 80 °C until use [10, 11]. The specific primers were designed to amplified complete sequence of the red-crowned crane parvovirus strain.(Table 1).

### Electron microscopic analysis

The DF-1 cells after five passages were used for electron microscopic analysis. Cell supernatants were centrifuged at 12,000 ×g for 5 min at 4 °C. Virus-containing supernatants were resuspended, negatively stained, and examined using transmission electron microscopy (TEM).

### Phylogenetic analysis

The complete genome of the red-crowned crane parvovirus strain was subjected to sequence alignments and phylogenetic analysis in comparison with the sequences of other 46 reference genomes from the Parvovirinae subfamily, and after the isolates were aligned with the reference genome using MAFF, the optimal model was analyzed using ModelFinder as GTR + F + R4. The maximum-likelihood tree was constructed using MEGA version 7.0, whose reliability was evaluated by the bootstrapping analysis with 1000 replicates, and the bootstrap value more than 50% was considered significant.

## Results and discussion

**Fig. 1:**
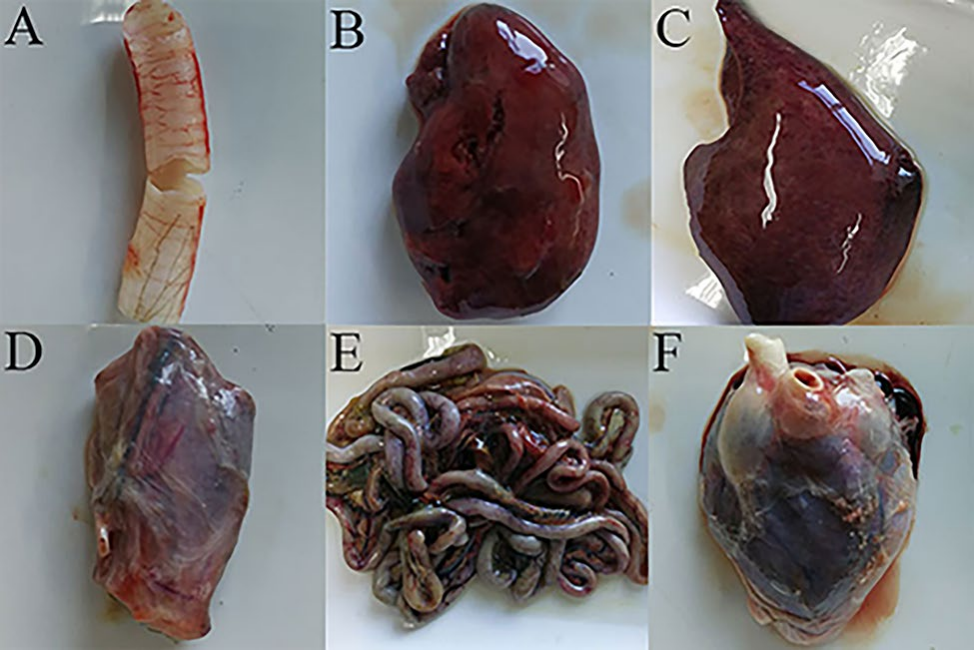
Diseased tissue collected from a dead, red-crowned crane. (**A**) Severely engorged trachea. (**B**) Severely engorged kidney. (**C**) Liver hemorrhage. (**D**) Esophageal engorgement. (**E**) Severe hemorrhage and edema in the intestinal tissue. (**F**) White nodules and swelling on the pericardium

**Fig. 2:**
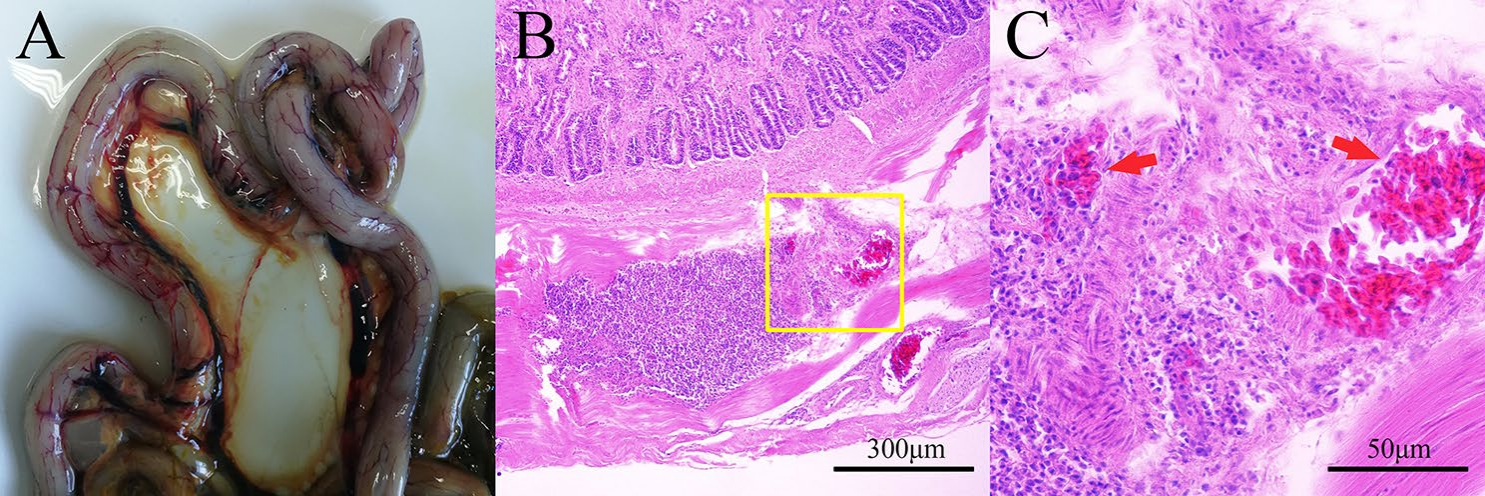
Histopathological examination of the intestines of a dead, adult, wild, red-crowned crane using HE staining. **A** Gross observation of the intestine indicated congestion occurred. **B** and **C** depicted the histopathology changes, and panel **C** (scale bar represents 50 μm) is amplified from panel **B** (scale bar indicates 300 μm). The structure of the crypt was damaged, and hemorrhage can be observed. The vascular epithelial cells significantly lose and inflammatory cells (lymphocyte and mononuclear macrophage) were found in the lumen of the vein

We dissected the red-crowned crane and observed tracheal, kidney, liver, esophageal and intestinal congestion as well as white nodules and swelling on the pericardium and intestinal lymph nodes (Fig. 1). Histopathological analysis confirmed that a large number of inflammatory cells infiltrated the intestine (Fig. 2). Using a metagenomic workflow, we identified 25 contigs of parvoviruses in pooled organ samples of the red-crowned crane. PCR indicated that the kidney, lung, heart, liver, and intestine were positive for parvovirus. However, the results of AIV, NDV, GPV, MDPV and ChPV were negative.

**Fig. 3:**
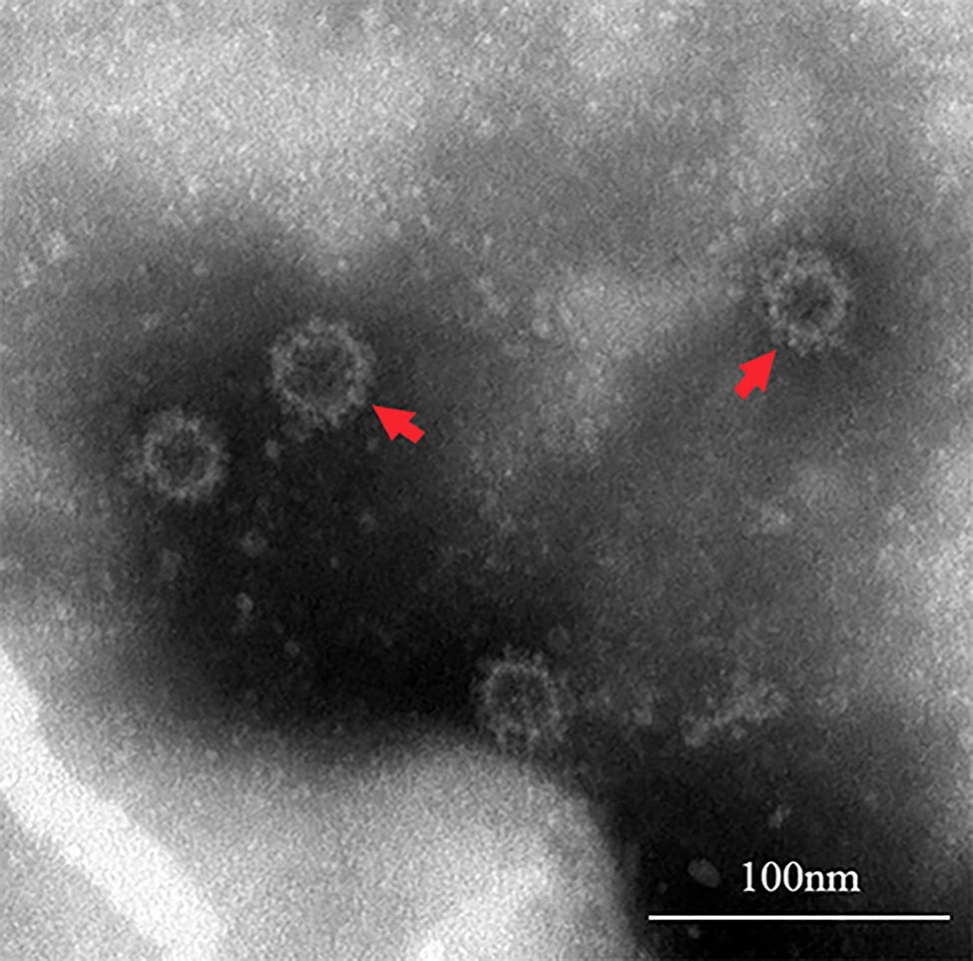
Electron microscopy of negatively stained parvovirus particles from the red-crowned crane. Scale bar indicates 100 nm

After the homogenate of dead red-crowned crane organs were inoculated in SPF chicken embryos were all chicken embryos survived but the vitality was weak in three days. The allantoic fluid were harvested and passaged three generations in SPF chicken embryos and then inoculate DF-1 cells. Cytopathogenic effects (CPEs) were consistently observed in DF-1 cells after 72 h. Then, the cells with CPEs underwent three cycles of freezing/thawing, the supernatant was collected and inoculated into DF-1 cells again. The above process was repeated for approximately five rounds until sufficient virus amplification. The supernatants containing parvovirus were resuspended and examined under a transmission electron microscope (TEM) after negative staining. TEM examination revealed spherical enveloped viral particles averaging 30 nm in diameter, a typical morphology of parvoviruses (Fig. 3).

**Fig. 4:**
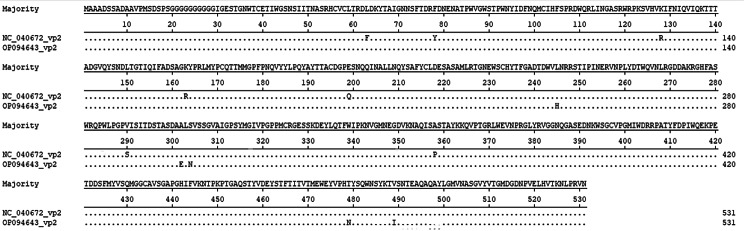
Aminoacid sequences of variable region in VP2 gene coding sequence.The sequence of isolate JL21/10 is indicated on the upper line. Only different amino acids from isolate JL21/10 are shown for yc8 strain

Subsequently, viral nucleic acids were extracted from the purified virus, and the complete genome of the parvovirus strain was obtained using PCR primers (Table 1). The genome of the red-crowned crane parvovirus JL21/10 strain (GenBank accession no. OP094643) contains 5,459 bp. Multiple sequence alignments of the complete genome of JL21/10 and other reference genomes from the parvovirus showed high identity of the red-crowned crane strain with parvovirus strains yc-8(Genbank Number: NC040672) at the nucleotide level (96.61%). Comparing individual proteins of JL21/10 with the yc-8 red-crowned crane parvoviruses, showed 92.5-99.4% amino acid identity with the nonstructural protein 1 (NS1) and nucleoprotein (NP), 97.7–98.2% with the major capsid protein (VP2) and minor capsid protein (VP1) (Table 2). For parvoviruses, the critical amino acid on the surface of the viral capsid, which interacts with the host receptor, probably determines its host range. The VP2 protein of the JL21/10 strain showed variations in 12 amino acids compared with the yc-8 strains(Fig. 4; Table 3).

**Table 2:** Amino acid sequence similarities of the JL21/10 strain with yc-8 sequences of red-crowned crane parvovirus strains

	NS1		NP		VP1		VP2	
Isolate	Length(aa)	Identity(%)	Length(aa)	Identity(%)	Length(aa)	Identity(%)	Length(aa)	Identity(%)
yc-8	680	92.5	161	99.4	672	98.2	531	97.7

**Table 3:** Sites of amino acid variations in the VP2 genes in the red-crowned crane parvovirus JL21/10 strain

Gene	Sites of amino acid variations compared with yc-8 strain
VP2	F63L,Y78F,R128K,R163K,Q199E,L245H,S290V,A302E,S304N,P358A,T479N,V489I

**Fig. 5:**
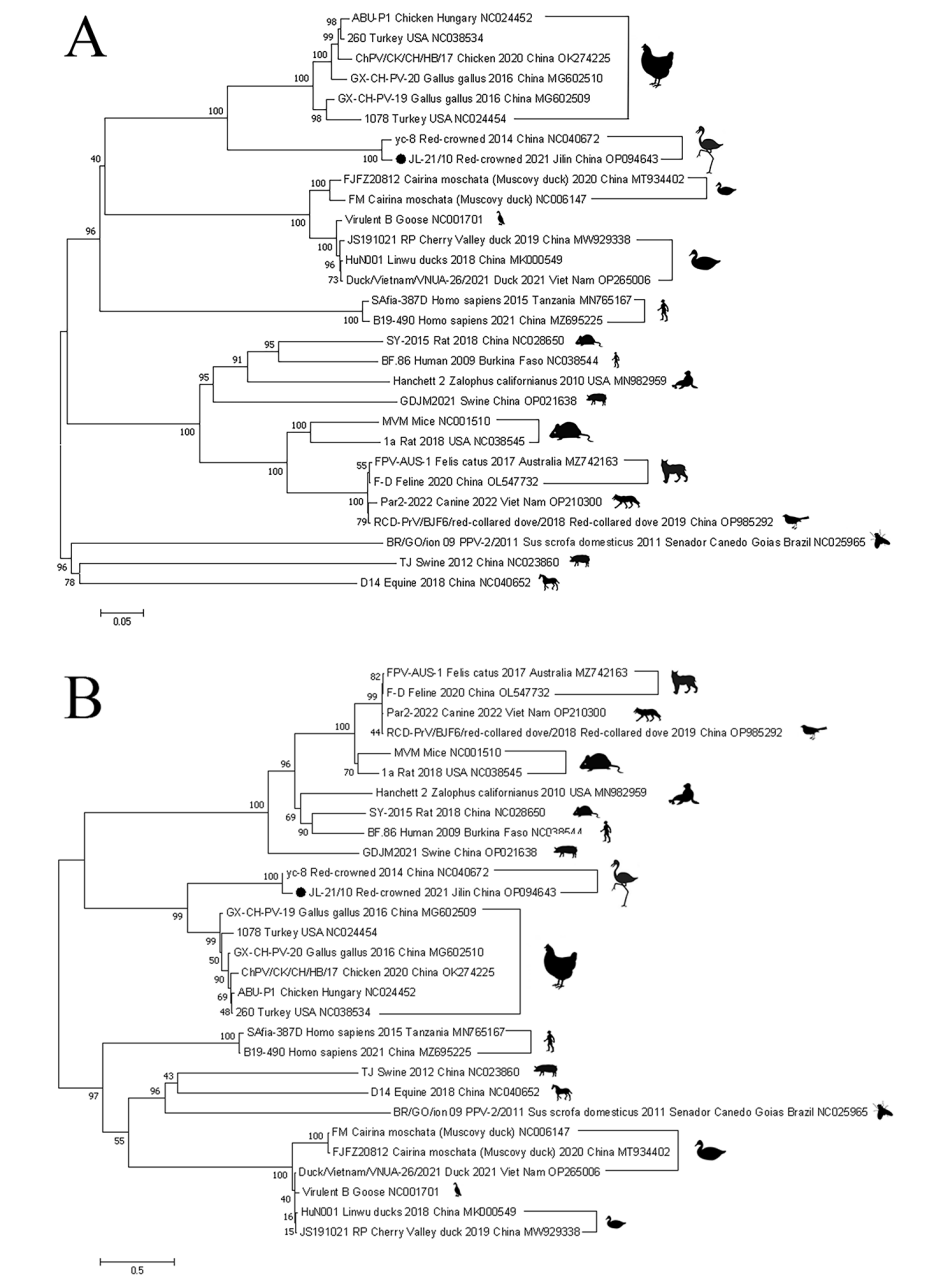
Phylogenetic analysis was carried out using 29 complete genome (**A**) and NS1 (**B**) gene of parvovirus strains. The JL21/10 strain identified in this study (GenBank accession number: OP094643) is labeled with a filled circle. The tree was generated using the maximum-likelihood model with MEGA. Bootstrap values were based on 1,000 replications

Phylogenetic analysis of the complete genome and NS1 gene revealed that the JL21/10 strain clustered with strains in poultry and revealed a close genetic relationship with the yc-8 strains (Fig. 5) [12].

The red-crowned crane is one of the rarest crane species, and its population is decreasing due to loss of habitat and viral infections [13–15]. However, there are only a few case reports of red-crowned crane mortality due to viral infection. In this study, we described a pathogenic virus causing severe diarrhea and serious tissue lesions in a red-crowned crane. Viruses can infect multiple organs and are highly pathogenic to intestinal tissue. We first used viral metagenomics and isolation to successfully isolate a novel parvovirus strain from the intestinal tissue of a dead, adult, wild, red-crowned crane. The whole genome sequence of the virus was obtained by PCR. Genetic analysis showed that JL21/10 strain was highly correlated with yc-8 strain of red-crowned crane parvovirus, but NS1, NP, VP1 and VP2 gene were found many gene mutation sites; The isolates of JL21/10 strain may be virulent with chicken as it infected chicken embryos and caused DF-1-cell CPEs. Therefore, we speculate that this virus might infect chicken or other birds.Further research on viral transmission and infection is urgently needed to protect red-crowned cranes and prevent cross-species transmission of the parvovirus to poultry, which would result in serious economic losses.

## Conclusion

In this study, we detected a parvovirus causing severe diarrhea in a red-crowned crane in China for the first time. Using PCR and histopathological analysis, we showed that parvoviruses can infect different organs and are highly pathogenic to intestinal tissue. Histopathological examination of the intestine showed that vascular epithelial cell significantly lose and inflammatory cells were found in the lumen of the vein. Blind passage experiment showed that red-crowned crane parvovirus had replication capability in SPF chicken embryos and DF-1.The virus could generate marked CPEs in cells within 3–4 days. The complete genome of the JL21/10 strain exhibited 96.61% nucleotide identities with those of yc-8 red-crowned crane parvoviruses. Phylogenetic analysis of the JL21/10 isolate showed clearly defined grouping into clusters with chicken and revealed a close genetic relationship with red-crowned crane. The amino acid homology of VP2 protein between JL21/10 and GX-CH-PV-19 strain of chicken was 55.4%.Whether the JL21/10 strain undergoes inter-species transmission between chicken and red-crowned crane remains an open question for further studies.

## Electronic supplementary material

Below is the link to the electronic supplementary material.

Supplementary Material 1

## Data Availability

All data and materials are within this published paper. The datasets generated and/or analysed during the current study are available in the NCBI GenBank database repository OP094643.

## Abbreviations

PCR: polymerase chain reaction
PPV: porcine parvovirus
CPV: canine parvovirus
FPV: feline parvovirus
GPV: goose parvovirus
MDPV: duck parvovirus
ChPV: chicken parvovirus
AIV: avian influenza virus
NDV: newcastle disease virus
SPF: specific pathogen-free
TEM: specific pathogen-free
CPEs: cytopathogenic effects
