# Supplementary material for: Isolation and characterization of a novel parvovirus from a red-crowned crane, China, 2021
Source: BMC Vet Res. 2023 Sep 21;19:169. doi: 10.1186/s12917-023-03683-4 (PMC10512598; doi:10.1186/s12917-023-03683-4)
Supplement: Supplementary file 1 — Supplementary Material 1 [file 12917_2023_3683_MOESM1_ESM.docx]

**A Novel Parvovirus from a Red-Crowned Crane, China, 2021**

**Technical Appendix**


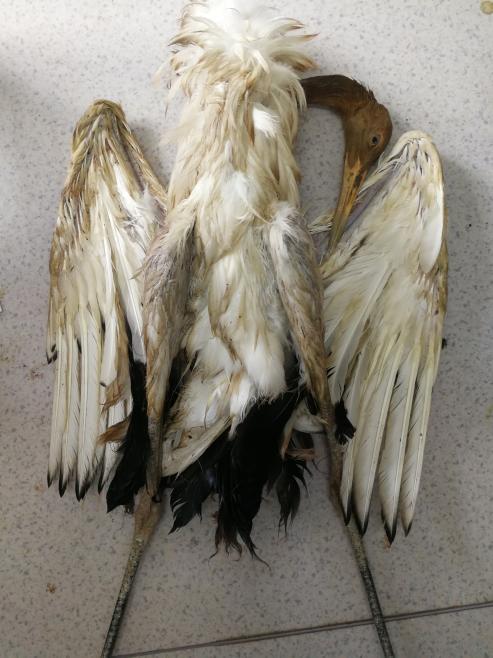


**Appendix Figure S1.** The adult, wild, red crowned crane presented with decreased food intake and bloody stools; over 5 days of treatment it lost weight and experienced sudden death.
